# GPR30 regulates diet-induced adiposity in female mice and adipogenesis *in vitro*

**DOI:** 10.1038/srep34302

**Published:** 2016-10-04

**Authors:** Aihua Wang, Jing Luo, William Moore, Hana Alkhalidy, Ling Wu, Jinhua Zhang, Wei Zhen, Yao Wang, Deborah J. Clegg, Bin Xu, Zhiyong Cheng, Ryan P. McMillan, Matthew W. Hulver, Dongmin Liu

**Affiliations:** 1 Departments of Human Nutrition, Foods and Exercise, College of Agricultural and Life Sciences, Virginia Tech, Blacksburg, VA, USA; 2 Departments of Human Nutrition, Foods and Exercise, and Biochemistry, College of Agricultural and Life Sciences, Virginia Tech, Blacksburg, VA, USA; 3 Biomedical Research Division, Diabetes and Obesity Research Institute, Department of Biomedical Science, Cedars-Sinai Medical Center, Los Angeles, CA, USA.

## Abstract

Recent studies showed that GPR30, a seven-transmembrane G-protein-coupled receptor, is a novel estrogen receptor (ER) that mediates some biological events elicited by estrogen in several types of cancer cells. However, its physiological or pathological role *in vivo* is unclear. Here, we show that GPR30 knockout (GPRKO) female mice were protected from high-fat diet (HFD)-induced obesity, blood glucose intolerance, and insulin resistance. The decreased body weight gain in GPRKO female mice is due to the reduction in body fat mass. These effects occurred in the absence of significant changes in food intake, intestinal fat absorption, triglyceride metabolism, or energy expenditure. However, GPR30 had no significant metabolic effects in male mice fed the HFD and both sexes of mice fed a chow diet. Further, GPR30 expression levels in fat tissues of WT obese female mice were greatly increased, whereas ERα and β expression was not altered. Deletion of GPR30 reduced adipogenic differentiation of adipose tissue-derived stromal cells. Conversely, activation of GPR30 enhanced adipogenic differentiation of 3T3-L1 preadipocytes. These findings provide evidence for the first time that GPR30 promotes adipogenesis and therefore the development of obesity in female mice exposed to excess fat energy.

GPR30 is a seven transmembrane G-protein-coupled receptor (GPCR)^1^. It is expressed in numerous tissues including reproductive systems, adipose tissue, vasculature, intestine, ovary, central nerve system, pancreatic islets, neurons, inflammatory cells, and bone tissue^2^. It has been shown that GPR30 induces signaling via activation of Gαs or Gαi^3^,^4^, strongly suggesting that the plasma membrane is the action site of this receptor. Intriguingly, while GPR30 is expressed in the plasma membrane^4^,^5^,^6^, a larger fraction of total cellular GPR30 can be located in intracellular compartments, including the endoplasmic reticulum and the Golgi complex^6^,^7^,^8^,^9^,^10^,^11^,^12^, suggesting that GPR30 may be an atypical GPCR. Indeed, studies show that GPR30 is activated intracellularly, which then diffuses across cell membranes and initiates cellular signaling^10^,^11^.

GPR30 is now recognized as a specific G-protein coupled estrogen receptor (ER) because it has a high affinity (nanomolar) for 17β-estradiol (E2)^4^,^11^. However, the physiological or pathological role of GPR30 is still unclear. Data from *in vitro* studies has demonstrated that GPR30 mediates some rapid biological events elicited by E2 in several types of cells that ultimately lead to cell proliferation and migration^13^,^14^,^15^,^16^,^17^,^18^. However, the biological relevance of these findings obtained from cultured cells is unclear. Indeed, recent studies showed that administration of G1, a specific GPR30 agonist^19^, did not stimulate estrogenic effects in the uterus and mammary gland of mice^9^. In contrast, studies from ovariectomized mice demonstrated that activation of GPR30 inhibits E2-induced uterine epithelial cell proliferation via inhibition of E2-stimulated ERα activity^20^. These results indicate that GPR30 may act as a negative regulator for some ER-mediated physiological processes.

It has been established that E2 plays a significant role in fat metabolism in both humans and rodents^21^,^22^,^23^,^24^. While classical ERs have been well investigated regarding their roles in mediating E2 effects on fat metabolism and metabolic diseases, the metabolic action of GPR30 is still unclear. It was showed that GPR30 deficiency caused a number of metabolic alterations and reduced body weight (BW) and bone growth in female, but not male mice, fed a standard chow diet (STD)^25^. On the contrary, another recent study showed that BW and abdominal adiposity were increased in both GPR30 knockout (GPRKO) male and female mice fed the STD^26^. Interestingly, Davis *et al.* reported that only male, but not female GPRKO mice displayed the significantly increased fat mass as compared to their wide-type (WT) littermates fed a STD^27^. However, several other studies reported no significant effect of GPR30 on BW of either female or male mice^28^,^29^. The reasons for these disparate results are not clear. However, most previous studies were not specifically designed for investigating the roles of GPR30 in obesity development in females. As female mice in these studies were used at their young ages and fed a STD during the experiments, they remain lean without apparent metabolic abnormalities, which therefore may be not sufficient to reveal the role of GPR30 in obesity development in females that is typically induced by high calorie intake. In this study, we investigated the metabolic effects of GPR30 in mice and its effect on adipogenesis *in vitro*.

## Results

### GPR30 deficient female mice are resistant to diet-induced obesity and glucose intolerance

**Figure 1:**
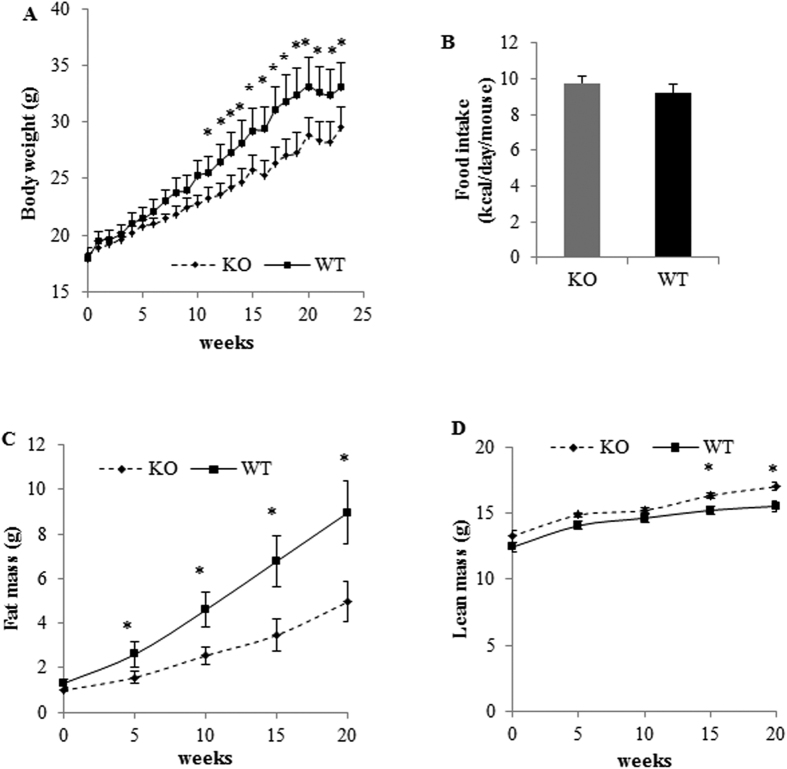
Deletion of GPR30 reduces adiposity in female mice fed a HFD. WT and KO female mice (12 wks old) with identical initial body weight were fed a HFD for 23 wks. Weekly body weight (**A**), food consumption as calorie intake (**B**), and fat (**C**) and lean (**D**) mass are shown. Data are mean ± SEM (n = 8 mice/group). *p < 0.05.

We examined GPRKO and WT mice either maintained on a chow diet (STD) or fed a high-fat diet (HFD) to determine the metabolic effects of GPR30. There were no differences in BW, fat mass, and all other measured metabolic phenotypes between GPRKO and WT female mice on a STD through the experiment (data not shown). However, when female mice were fed a HFD, the BW between the GPRKO and WT mice became significantly different after 12 wks. After 23 wks on HFD, the BW of WT female mice increased by 85%, whereas GPR30 KO females gained 61% of their starting BW (p < 0.05) (Fig. 1A). The amount of food intake however was similar between female WT and KO mice (Fig. 1B), indicating that the lower BW of KO mice was not due to reduced caloric intake. Data of NMR-based body composition analysis show that fat mass in WT and GPRKO females was similar before exposed to HFD, but became significantly different after 5 wks on HFD (Fig. 1C). The fat mass in GPRKO female mice relative to WT mice became more diverged with advancing age. After exposure to HFD for 20 wks, fat mass in WT mice (8.97 g) was 1.8 fold of that in GPRKO mice (4.97 g) (Fig. 1C), but GPRKO mice only had slightly higher lean body than that of the control mice (Fig. 1D). Therefore, the difference in BW between WT and KO mice were primarily due to their fat mass difference. As this is the first time showing, to the best of our knowledge, that deletion of GPR30 reduces adiposity of HFD-fed female mice, we conducted second study with another cohort of female mice and obtained the similar results (data not shown). Interestingly, deletion of GPR30 had no effect on metabolic phenotypes in male mice fed a HFD (Supplemental Figure 1). Interestingly, there was no difference in GPR30 gene expression in white adipose tissue (WAT) between male and female mice (Supplemental Figure 2).

**Figure 2:**
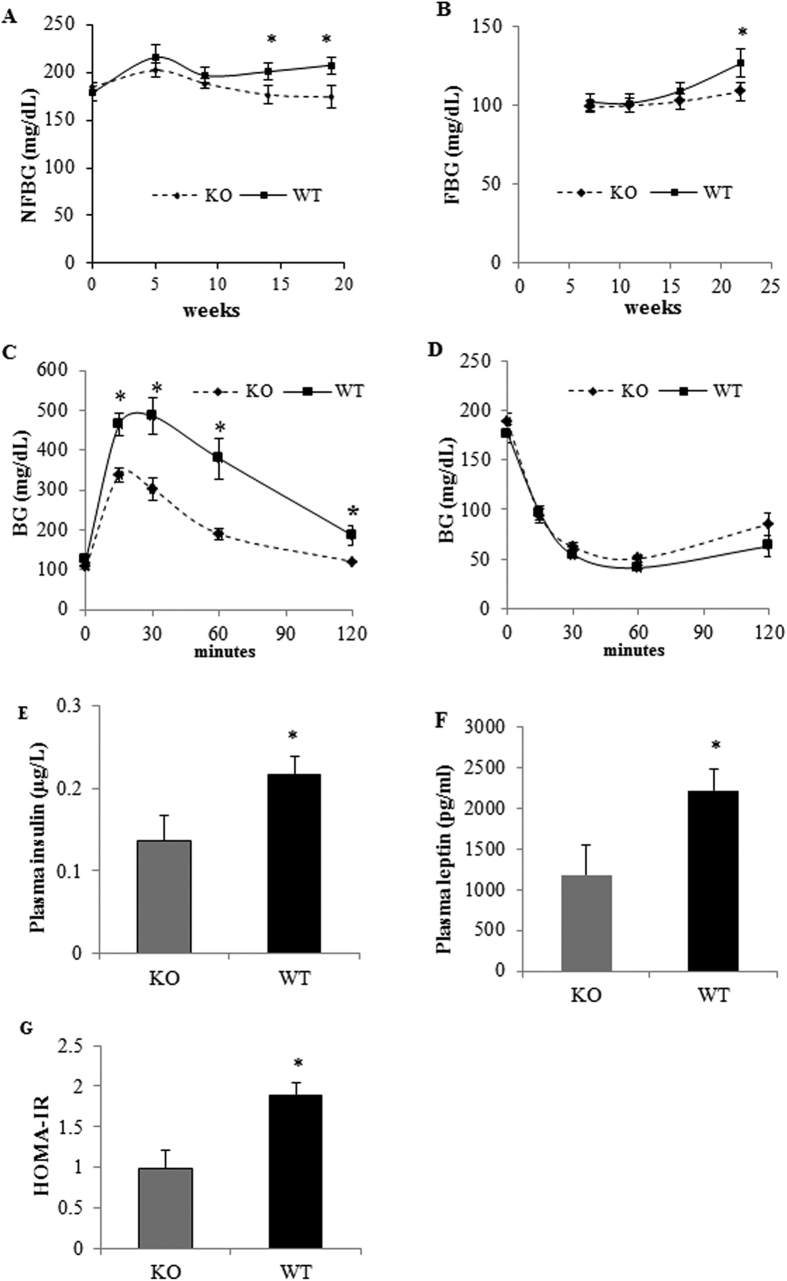
Deletion of GPR30 improves glucose homeostasis and insulin sensitivity in female mice fed a HFD. (**A)** Non-fasting blood glucose (NFBG) and (**B)** Fasting blood glucose (FBG) levels were measured at indicated weeks of HFD feeding. Glucose (**C**) and insulin (**D**) tolerance tests were determined at 22 and 23 wks, respectively. Plasma insulin (**E**) and leptin (**F**) levels in overnight fasted mice after 23 wks on HFD were measured by enzyme immunoassay kits. HOMA-IR was calculated as stated in the “Methods” section (**G**). Data are mean ± SEM (n = 8 mice/group). *P < 0.05.

To determine if GPR30 affects glucose homeostasis, we measured NFBG at 0, 5, 9, 14, 19^th^ wks and FBG concentrations at 7, 11, 16, 22^nd^ wks. GPRKO mice fed HFD displayed significantly lower non-fasting blood glucose (NFBG) levels as compared with those in WT mice (Fig. 2A). Fasting blood glucose (FBG) levels of HFD-fed GPRKO and WT mice gradually diverged after 15 wks and by 22^nd^ wk, GPRKO females on a HFD were 14% lower in FBG concentrations as compared with WT females (p < 0.05; Fig. 2B). Consistently, Female GPRKO mice were more glucose tolerant than WT mice (Fig. 2C). While whole body insulin sensitivity was not different between WT and GPRKO mice (Fig. 2D), WT female mice fed a HFD had higher insulin (Fig. 2E) and leptin (Fig. 2F) levels than those in GPRKO female mice (p < 0.05; Fig. 2E), which are typically associated with obesity and insulin resistance. HFD-fed GPRKO female mice were 52% lower in homeostatic model assessment of insulin resistance (HOMA-IR) than that of WT female mice (p < 0.05) (Fig. 2G).

### Deletion of GPR30 has no effect on fat metabolism or postprandial triglyceride clearance

**Figure 3:**
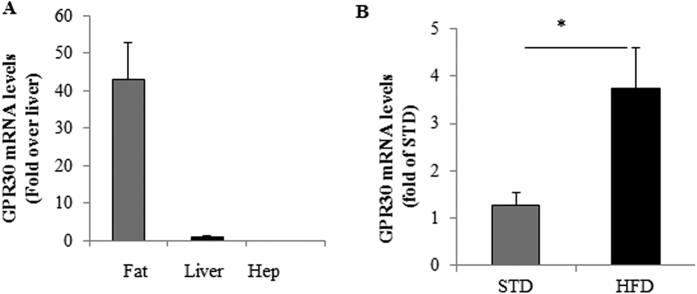
GPR30 mRNA expression was absent in liver hepatocytes but upregulated in WAT of female mice by HFD feeding. (**A**) GPR30 gene expression in fat tissue, liver, and primary hepatocytes (Hep) of the mice was measured by quantitative real-time RT-PCR. GPR30 mRNA expression in adipose tissue from female mice fed STD or HFD was also determined (**B**). Data are mean ± SEM (n = 8 mice/group). *p < 0.05.

To determine if GPR30 deficiency reduces intestinal fat absorption, thereby causing the decrease in fat deposit, we collected feces at 10^th^ and 19^th^ wk of HFD treatment and measured triglyceride content. No significant differences in fecal triglyceride levels were observed between WT and GPRKO female mice (Supplementary Figure 3A). In addition, we didn’t found significant difference in fat tolerance between GPRKO and WT mice (Supplementary Figure 3B). Consistently, liver triglyceride contents were similar between WT and GPRKO mice (Supplementary Figure 1C) as well as the gene expression levels of transcription factors ChREBP and SREBP-1c (data not shown), which coordinate the expression of genes required for fatty acid synthesis (20, 21). These results indicate that deletion of GPR30 has no effect on postprandial hepatic triglyceride metabolism and plasma clearance of intragastrically loaded triglycerides. Both WT and GPRKO female mice fed HFD had similar fasting plasma total cholesterol (Supplementary Figure 3D) and non-esterified fatty acids (NEFA; Supplementary Figure 3E) levels, suggesting that lipolysis may not be altered by deletion of GPR30. Paradoxically, KO mice displayed higher triglyceride concentrations as compared with WT mice (Supplementary Figure 3F). We then analyzed GPR30 gene expression in fat tissue, liver, and primary hepatocytes of the mice. We found that GPR30 mRNA was barely detectable in the liver and was completely absent in isolated mouse hepatocytes, but it was highly expressed in WAT of WT mice (Fig. 3A). Interestingly, GPR30 mRNA abundance in WAT of female mice was greatly up-regulated by HFD feeding (Fig. 3B). Collectively, these results provide strong evidence that WAT but not liver or intestine is the primary site for GPR30 to regulate adiposity in HFD-fed female mice.

### GPR30 deficiency reduces fat depot mass and adipocyte size

**Figure 4:**
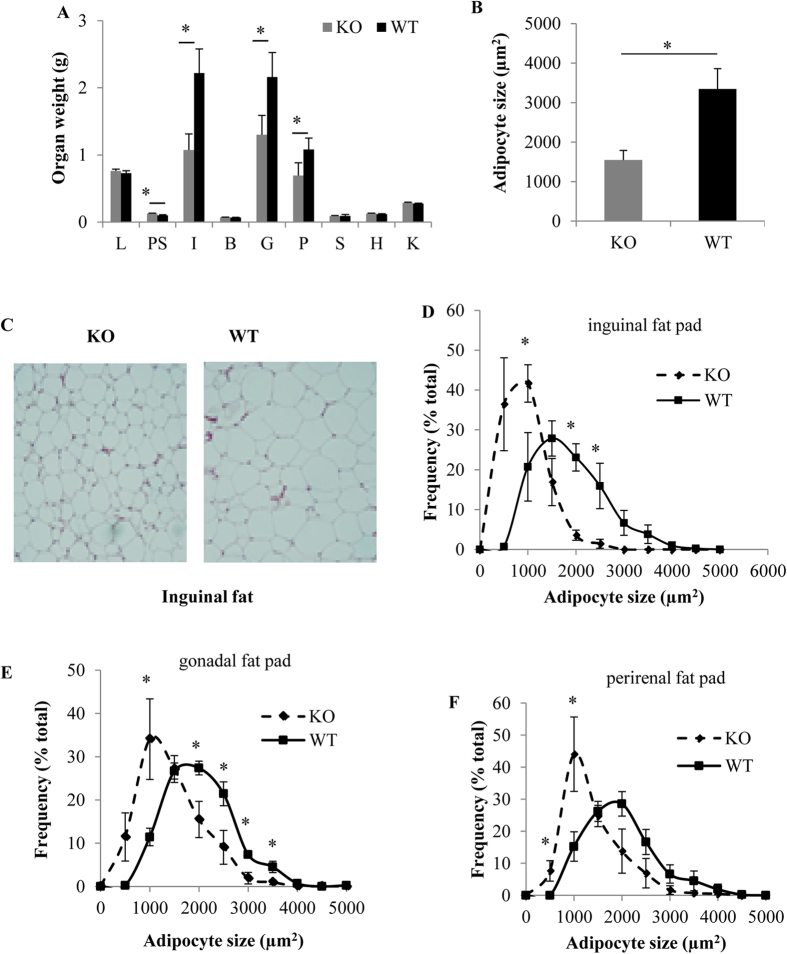
GPR30 KO reduces fat mass and adipocyte size in female mice fed a HFD. (**A**) Fat pad and organ weights of female mice fed a HFD for 23 wks. (**B**) Average adipocyte size of inguinal fat. (**C**) Representative images from inguinal fat tissue. The distribution of different sizes of adipocytes in inguinal (**D**), gonadal (**E**) and perirenal **(F)** fat pads. Data are mean ± SEM (n = 8 mouse/group). *p < 0.05. **Note**: L = liver, PS = pancreas, I = inguinal fat, B = brown fat, G = gonadal fat, P = perirenal fat, S = spleen, H = heart, K = kidney.

To gain insight into alterations of body composition, we euthanized mice after 23 wks of HFD treatment and collected various fat pads and organs. The inguinal, gonadal, and perirenal fat pads from GPRKO mice weighted significantly less than those in WT mice (Fig. 4A). There were no differences in brown fat mass and other organs except pancreas between WT and GPRKO mice. In addition, ectopic lipid accumulation in muscle, heart, aorta, and kidney was not observed in both genotypes. These results suggest that the reduced fat mass in GPRKO female mice fed HFD is not a result of decreased body growth. During the development of obesity, adipose tissue undergoes hyperplasia as well as hypertrophy for the increased demand for triglyceride storage^30^,^31^. In order to determine if the differences in fat mass between KO and WT mice was due to differences in adipocyte size, we performed H&E staining of fat sections, which labels adipocyte plasma membranes, allowing for adipocyte size measurements in paraffin sectioned WAT to evaluate the contribution of adipocyte hypertrophy to WAT mass accumulation. We found that GPRKO female mice had smaller adipocytes as compared to WT female mice (Fig. 4B,C). Further analyses of adipocyte size distribution revealed that GPR30 deficiency caused the shift toward smaller adipocytes cross fat pads (Fig. 4D–F).

### Deletion of GPR30 has no effects on plasma E2 level and expression of ER and adipogenic factors in WAT

Because GPR30 is an ER^4^,^11^, we wondered whether deletion of GPR30 in female mice affects circulating E2 levels and classical ER expression in WAT. We found that neither plasma E2 levels (Supplementary Figure 4A) nor ERα and ERβ gene expression were altered by deletion of GPR30 (Supplementary Figure 4B), suggesting that GPR30 effect on adiposity was not due to the secondary action whereby its absence modulated E2 production or ERα/β expression. In addition, deletion of GPR30 had no significant effect on gene expression of several adipogenic transcription factors in WAT, including peroxisome proliferator-activated receptor-γ (Pparγ), CCAAT/enhancer binding protein-α (Cebpα), Cebpβ, C/ebpδ, and bone morphogenetic protein 2 (Bmp2) (Supplemental Figure 4C).

### The effects of GPR30 on body temperature, energy expenditure, and fatty acid oxidation (FAO)

**Figure 5:**
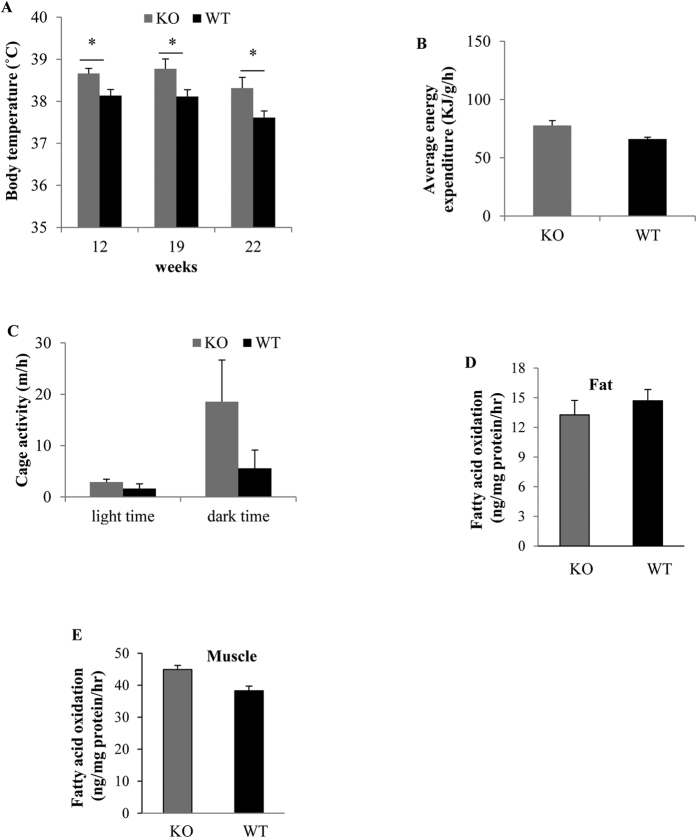
GPR30 deficiency increased body temperature but had no significant effect on energy expenditure and fatty acid oxidation. Female WT or KO mice were fed a STD or HFD for 23 wks. Rectal body temperatures were measured at 12, 19 and 22^nd^ wk (**A**). The average energy expenditure (**B**) and cage activity during light time and dark time (**C**) were measured after 23 wks of treatment. Fatty acid oxidation in white adipose tissues (**D**) and skeletal muscle (**E**) from WT and KO female mice were also determined. Data are mean ± SEM (n = 8 mice/group). *p < 0.05.

As the absence of GPR30 didn’t alter the amount of food intake in mice, we examined if deletion of GPR30 increased energy expenditure, thereby resulting in reduced fat deposits. In this regard, we first measured body temperature at 12, 19 and 22 wks of treatment. Female GPRKO mice fed HFD had significantly higher rectal body temperature than that of HFD-fed WT female mice (38.7, 38.8, and 38.3 °C for KO mice vs 38.1, 38.1, and 37.6 °C for WT mice at 12, 19, and 22 wks, respectively) (Fig. 5A). GPRKO female mice tended to have higher energy expenditure than the WT female mice (Fig. 5B), but the difference was not statistically significant (p = 0.07). In addition, female GPRKO mice fed HFD tended to be more active than WT female mice during dark time (p = 0.0825) (Fig. 5C). Further, absence of GPR30 didn’t alter FAO in WAT (Fig. 5D) or muscle (Fig. 5E).

### GPR30 regulates adipogenesis of mouse adipose-derived stromal cells and 3T3-L1 preadipocytes

**Figure 6:**
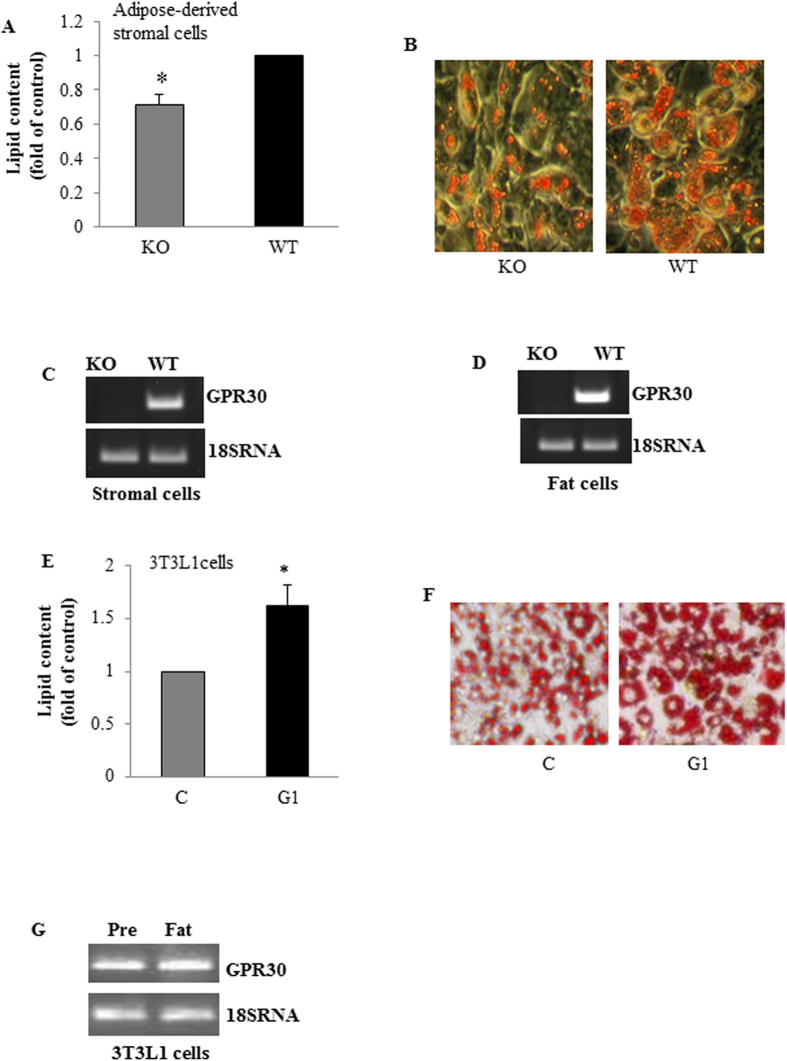
GPR30 regulates adipogenesis. Adipose-derived stromal cells from GPRKO or WT female mice were cultured in differentiation medium for 6 days. (**A)** The differentiated cells were visualized by staining with Oil Red O and stained lipids were extracted and quantified. (**B)** Shown are representative images from four experiments. GPR30 gene expression was measured in WT and KO adipose-derived stromal cells (**C**) and differentiated fat cells from stromal cells (**D**) by RT-PCR (a representative full-length gel image is included in the Supplementary Information). Post-confluent 3T3-L1 cells were incubated in differentiation medium with or without G1 (100 nM) as described in “Materials and Methods”. Lipid accumulation in the differentiated cells were measured (**E**), and representative images of Oil Red O staining of intracellular lipids from three experiments in triplicated determinations each are shown (**F**). (**G**) GPR30 gene expression in 3T3-L1 preadipocytes (Pre) and fully differentiated fat cells (fat) as analyzed by RT-PCR. Data are mean ± SEM. *p < 0.05 *vs.* control.

We further examined whether GPR30 directly regulates adipogenesis, which is crucial in driving the expansion of adipose tissue mass that leads to obesity. Consistent with the reduced fat mass in GPRKO mice, adipose-derived stromal cells from GPRKO displayed a significantly lower rate of differentiation as compared with WT cells (Fig. 6A,B). We further confirmed that GPR30 gene was expressed in WT stromal cells (Fig. 6C) and their differentiated fat cells (Fig. 6D), but was completely absent in cells from GPRKO mice (Fig. 6C,D). Consistently, activation of GPR30 by G1 increased adipogenesis of 3T3-L1 cells (Fig. 6E,F) without affecting cell proliferation (data not shown). We further show that GPR30 mRNA is abundantly present in both 3T3-L1 preadipocytes and fully differentiated adipocytes (Fig. 6G). These data suggest that the reduced adiposity in GPRKO female mice may be due to decreased adipogenesis.

## Discussion

In the present study, we found that deletion of GPR30 protected female mice from developing obesity, glucose intolerance, and insulin resistance when challenged with a HFD. Interestingly, all these effects are not observed in male mice. We also analyzed GPR30 mRNA levels in adipose tissues of male and female mice, and found that there was no significant gender difference in adipose expression of GPR30. These data demonstrate that GPR30 regulation of adipose tissue energy metabolism in response to HFD exposure is female-specific and may be E2-dependent. While data from the present study show that GPRKO female mice fed the HFD displayed better insulin sensitivity and glucose homeostasis, these beneficial effects may be the secondary effects whereby deletion of GPR30 prevented obesity in mice fed a HFD, given that deletion of GPR30 had no effects on blood glucose, insulin, and insulin sensitivity in STD-fed mice. However, we can’t exclude the possibility that GPR30 may directly modulate glucose metabolism in HFD-fed mice that could lead to the improved glucose tolerance in HFD-fed mice.

As GPR30 is not involved in regulating calorie intake in mice, we then addressed whether GPR30 affects energy expenditure or ambulatory activity, which can contribute to the reduced body fat mass. It is worth noting that in the present study energy expenditure was normalized to lean mass instead of BW, because fat tissue may contribute comparatively less to the total energy expenditure compared with lean mass due to its relatively low metabolic activity^32^. While KO female mice fed with HFD tended to have higher energy expenditure and cage activity than those of WT female mice, the differences didn’t reach statistical significance. Consistently, deletion of GPR30 had no significant effect on fatty acid oxidation in WAT and skeletal muscle, suggesting that the ability of mitochondria to oxidize fatty acids in these tissues was not altered by GPR30. However, there is possible that the small difference in increased daily energy expenditure and physical activity between WT and KO mice could lead to the large differences in the accumulation of fat mass over time^32^,^33^. BAT plays a critical role in maintaining body temperature and balancing energy expenditure in mammals^34^. We observed that GPRKO mice had higher body temperature, suggesting that GPR30 could regulate fatty acid^35^,^36^ and/or glucose^37^,^38^ metabolism in BAT, which dissipates energy from glucose and fatty acids into heat^39^. This aspect needs to be further investigated.

Disruption of fat digestion and absorption attends HFD-induced obesity. However, this is not the case for GPRKO mice, as the fecal triglyceride and NEFA contents were similar between fed WT and KO mice. Increased secretion of VLDL companied with disturbed clearance of triglycerides contributes to the development of obesity. In the present study, neither fat tolerance nor NEFAs levels in the blood between KO and WT mice were different. In addition, we believe that GPR30 is not involved in regulating de novo lipogenesis and triglyceride secretion in the liver, as deletion of GPR30 did not alter hepatic triglyceride contents and gene expression of transcription factors CHREBP and SREBP-1c, which coordinate the expression of genes required for fatty acid synthesis. Indeed, GPR30 mRNA was barely detectable in the liver and was absent in isolated mouse hepatocytes, while it was highly expressed in WAT of HFD-fed WT female mice. Interestingly, GPRKO mice had significantly higher fasting plasma triglyceride levels than those in WT mice. The reason for this difference is unclear. It is possibility that WT mice may be able to accumulate more triglycerides in the fat tissues given that WT mice fed a HFD had larger adipocytes as compared with those in KO mice. In addition, deficiency of GPR30 could affect plasma lipoprotein lipase activity during fasting, thereby modulating triglyceride levels. Nevertheless, these results provide strong evidence that the reduced fat accumulation by deletion of GPR30 is not due to altered fat absorption, hepatic lipid metabolism, or lipoprotein lipase-mediated triglyceride clearance.

While how exactly GPR30 regulates adipose tissue fat metabolism is still unclear, our data demonstrate that the effect of GPR30 on fat mass in HFD-fed female mice was not due to a secondary action by which its deletion altered circulating E2 levels or expression of ERα, which is believed to play a major role in mediating estrogenic effects on energy homeostasis^40^. Both human and rodent WAT expresses ERα, ERβ, and GPR30, suggesting that E2 signaling could occur through both ERs and GPR30. Interestingly, it was reported that GPR30 and ERα inhibit each other’s actions in several types of cells^7^,^20^,^41^. These data suggest that there may be a “ying-yang” relationship between GPR30 and ERα in regulating energy metabolism in adipose tissue in response to E2. In that regard, activation of ERα by E2 inhibits adiposity, whereas activation of GPR30 might promote obesity. It was demonstrated that plasma E2 levels were increased in ERα KO female mice^42^, which could lead to increased E2/GPR30 signaling. If this is true, it is possible that HFD-induced obesity in ERα KO female mice could be at least partially due to enhanced E2/GPR30 signaling in the lack of ERα, an aspect that is currently under investigation in our lab.

Adipogenesis plays an important role in the expansion of WAT mass that leads to obesity. WAT-derived stromal cells is a rich source of preadipocytes and mesenchymal stem cells that can be induced to differentiate into adipocytes^43^. We found that deletion of GPR30 reduced adipogenic differentiation of WAT-derived stromal cells. Consistently, activation of GPR30 increased adipogenesis of 3T3-L1 preadipocytes. These data, along with our *in vivo* finding that GPR30 expression was upregulated in mice fed HFD, suggest for the first time that GPR30 may play a role in promoting obesity in females by at least partially acting in WAT to regulate adipogenesis. It is presently unknown however how GPR30 regulates adipogenesis, given that deletion of GPR30 had no significant effects on the gene expression of several important adipogenic factors in WAT. Fatty acid synthase (FAS) is a key lipogenic enzyme that catalyzes the generation of palmitate from malonyl-CoA and acetyl-CoA^18^. FAS is also expressed in WAT. Recently studies showed that adipose FAS plays an important role in adipogenesis and obesity development^44^. It was recently shown that activation of GPR30 by E2 increases FAS gene expression in cancer cells^18^. It is therefore possible that FAS may be the downstream target of the GPR30 signaling pathway that mediates its adipogenic action, which is presently under investigation in our laboratory.

In summary, the present study provides evidence for the first time that GPR30 promotes adipogenesis and thereby obesity in mice exposed to excess fat energy. The HFD-induced increase in GPR30 expression in WAT may lead to increased E2/GPR30 signaling, which could counteract the role of ERα in regulating adipose tissue energy homeostasis, an aspect that need further investigation.

## Methods

### Animals

GPR30 heterozygous mice on 129 background were kindly provided by Dr. Deborah J. Clegg (UT Southwestern Medical Center, TX). Homozygous GPRKO and their littermate WT mice were generated by matting heterozygous mice and genotyped using quantitative RT-PCR. All mice were housed under constant temperature (23–24 °C) with a 12-h light/dark cycle and *ad libitum* access to food and water. The BW of GPRKO and WT mice was similar at weaning (3–4 wks) and thereafter when exposed to a STD. At 12 wks old, female mice were divided into 4 groups with 7–8 mice per group and fed either a STD with 18% of calories from fat or a HFD (Research Diets Inc., NJ) with 58% of calories from fat for 23 wks. Food intake and BW were recorded weekly. The weights of major organ including fat pads were recorded after mice were euthanized. For comparing GPR30 gene expression in WAT between male and female mice, WT female mice and their male littermates fed a STD were euthanized at 18 wks old, and the gonadal fat tissues were then collected for this analysis. All animal studies were approved by the Institutional Animal Care and Use Committee (IACUC) at Virginia Tech, and all experiments were strictly carried out in accordance with approved protocols and regulations by IACUC.

### Body composition and energy expenditure measurements

Body composition of the mice was evaluated using a nuclear magnetic resonance-based instrument (Bruker Optics Inc, MA) at 0, 5, 10, 15, and 20 wks of the feeding experiment. Body temperature was measured using a thermometer probe placed at a 2.5 cm depth in the rectum. After 23 wks of treatment, mice were transferred to metabolic cages for assessing energy expenditure and voluntary cage activity using an indirect calorimetry system (TSE Systems, Inc, MO)^45^. The rates (ml/kg/h) of oxygen consumption (VO_2_) and carbon dioxide production (VCO_2_) for each mouse were recorded at 20-min intervals for 48 h. Total energy expenditure was calculated as EE = VO_2_ × (3.815 + (1.232 × RER)) and normalized to body lean mass (kcal/kg/h). Home cage activity was recorded at 20-min intervals for 48h and expressed as total distance moved per hour.

### Measurement of adipocyte area and size

The inguinal, gonadal, and perirenal adipose tissues were fixed in 10% of phosphate buffered formalin for 18h, dehydrated, and embedded in paraffin. Tissues were then sectioned and stained with hematoxylin and eosin (H/E, Fisher Scientific, PA) and photographed at 10× of magnification. Areas of adipocytes were measured using the ImageJ software (NIH, MD). At least 100 adipocytes were counted from each section and 3 sections from each mouse. The frequency distribution of adipocyte sizes in fat depots was quantified as described^46^. Briefly, Data were loaded into a spreadsheet of Microsoft Excel program, and the number of adipocytes within the distribution from 0 to 5,000 μm^2^ with 500 increment was then calculated using the frequency function. The frequency distribution of adipocyte sizes was expressed as a percentage of total adipocytes counted.

### Measurements of fatty acid oxidation (FAO) in white adipose tissue (WAT) and skeletal muscle

Mice were fasted overnight and gonadal fat tissues and skeletal muscle from the gastrocnemius and quadriceps were collected after euthanization. Tissue homogenates were incubated in buffer containing [1-^14^C] palmitic acid. FAO was assessed by measuring and summing ^14^CO_2_ production and ^14^C-labeled acid-soluble metabolites from the oxidation of [1-^14^C] palmitic acid (American Radiolabeled Chemicals, MO)^47^.

### Fecal triglyceride analysis

Feces were collected for 3 consecutive days at 10 and 19 wks of feeding experiment. Fecal lipids were extracted as previously described^48^. Briefly, feces were weighed and lipids were extracted with choleroform-methanol (2:1). After centrifugation, chloroform phase was collected and lipid extracts were dried under a stream of nitrogen gas and then resuspended in chloroform-1% Triton X-100. The samples were evaporated again and finally dissolved in ddH_2_O. Total triglyceride concentrations were then measured using an assay kit (Teco Diagnostics, CA) and normalized to the dry weight of feces.

### Fat tolerance test and triglyceride measurement in the liver

To perform the fat tolerance test, mice were fasted overnight before administration of 10 μl/g BW of olive oil by gavage. Plasma triglyceride levels were measured at 0, 1, 2, 3, 4 and 8 h after oil administration. Liver triglyceride content was determined as reported^49^. The liver triglyceride contents are expressed as mg of triglyceride per gram of the liver sample.

### Blood chemistry

The fasting (FBG) and non-fasting (NFBG) blood glucose levels were measured at various time points throughout the experiment^50^. Plasma total cholesterol, triglyceride and non-esterified fatty acid (NEFA) were measured by using enzymatic assay kits (Teco Diagnostics, CA; Wako Diagnostics, CA). Plasma insulin levels were measured by ELISA (Crystal Chem, IL). Homeostasis model assessment of insulin resistance (HOMA-IR) was calculated using the following equation: HOMA-IR = (fasting plasma insulin (mU/l) × fasting plasma glucose (mmol/l))/22.5. Plasma E2 and leptin concentrations were determined by ELISA (Caymen Chemical, MI) and RIA (SPIbio, Montigny le bretonneux, France), respectively.

### Glucose and insulin tolerance tests

For intraperitoneal glucose tolerance test, mice were fasted for 12 h and then injected intraperitoneally with a single bolus of glucose (1 g/kg body weight). For insulin tolerance test, mice were fasted for 4 h and then intraperitoneally injected with human insulin (0.75 units/kg BW; Eli Lilly, IN). Glucose levels were measured post injection using a glucometer^50^.

### Isolation of hepatocytes

Mouse hepatocytes were isolated as previously described^51^. Hepatocytes were cultured with DMEM medium overnight before used for RNA extraction.

### Quantitative real-time RT-PCR

Total RNA was extracted from tissues with TRI reagent (Molecular Research Center, OH) and reverse-transcribed using GoScript™ Reverse transcriptase and random primers (Promega, WI). Amplification reactions were performed on an Applied Biosystems® 7500 Fast Real-Time PCR System as we previously described^52^. Data were analyzed by the 2^−ΔΔCt^ method. The primers used are: GPR30 (5′-TCATTTCTGCCATGCACCCA-3′ and 5′-GTGGACA-GGGTGTCTGATGT-3′), ERα (5′-CTGTCGGCTGCGCAAGTGTT-3′ and 5′-CATCTCTCTGACGCTTGTGCT-3′), ERβ (5′-GCCAACCTCCTGATGCTTCT-3′ and 5′-TCGTACACCGGGACCACAT-3′), Chrebp (5′-CTGGGGACCTAAACAGGAGC-3′ and 5′-GAAGCCACCCTATAGCTCCC-3′), Srebp-1c (5′-GATCAAAGAGGAGCCAGTGC-3′ and 5′-TAGATGGTGGCTGCTGAGTG-3′), Pparg (5′-ATTGAGTGCCGAGTCTGTGG-3′ and 5′-GCAAGGCACTTCTGAAACCG-3′), Cebpa (5′-AGCAACGAGTACCGGGTACG-3′ and 5′-TGTTTGGCTTTATCTCGGCTC-3′), Cebpb (5′-CGCAACCTGGAGACGCAGCA-3′ and 5′-GGCTCGGGCAGCTGCTTGAA-3′), Bmp2 (5′-GACTGCGGTCTCCTAAAGGTCG-3′ and 5′-CTGGGGAAGCAGCAACACTA-3′), 18S RNA (5′-ACCT GGTTGATCCTGCCAGTAG-3′ and 5′-TTAATGAGCCATTCGCAGTTTC-3′).

### Adipogenesis analysis

Stromal vascular cells from female WAT of WT and GPRKO mice were isolated as previously described^53^. The cells were grown to confluence in complete DMEM medium containing 10% FBS and then were cultured in adipocyte differentiation cocktail containing 5 μM dexamethasone, 2.5μg/ml insulin, 0.5 mM 3-isobutyl-1-methylxanthine (IBMX), 1nM T3^54^. After 2 days, cells were cultured in DMEM medium supplemented with 1.5 μg/ml insulin and 1 nM T3 for 4 days. 3T3-L1 preadipocytes were cultured and differentiated as we previously described^55^. Briefly, post-confluent cells were incubated in complete DMEM containing 1 μM dexamethasone, 0.5 mM IBMX, 1 μg/ml insulin with or without 100 nM G1 for 2 days. The cells were then washed with PBS and cultured in complete DMEM supplemented with 1 μg/ml insulin for additional 2 days. Afterwards, cells were maintained in DMEM medium for 8 days with medium changed every other day. The differentiated cells were visualized by Oil Red-O staining of intracellular lipids. Oil red-O stain in the cells was extracted with isopropanol and quantified using a microplate reader^55^.

### Statistical analysis

Data were analyzed with one-way ANOVA or the student’s t-test using JMP software (SAS Inc., NC), where appropriate. Values are expressed as means ± SEM. Treatment differences were subjected to t-test or Tukey’s test. A *P* < 0.05 was considered significant. Real-time PCR data were analyzed using the ∆∆C_T_ method, where 18S RNA served as the endogenous control and fat from control mice served as the calibrator sample. The ΔC_T_ = C_T target gene_–C_T 18S_, and ΔΔC_T_ = ΔC_T target sample_–ΔC_T calibrator_^56^. Relative quantities, calculated as 2^−∆∆CT^, were used for statistical analysis.

## Additional Information

**How to cite this article**: Wang, A. *et al.* GPR30 regulates diet-induced adiposity in female mice and adipogenesis *in vitro. Sci. Rep.* **6**, 34302; doi: 10.1038/srep34302 (2016).

## Supplementary Material

Supplementary Information
