# Supplementary material for: GPR30 regulates diet-induced adiposity in female mice and adipogenesis in vitro
Source: Sci Rep. 2016 Oct 4;6:34302. doi: 10.1038/srep34302 (PMC5048424; doi:10.1038/srep34302)

**Title:** GPR30 regulates diet-induced adiposity in female mice and adipogenesis in vitro

**Authors**: Aihua Wang[1*], Jing Luo[1*], William Moore[1], Hana Alkhalidy[1], Ling Wu[2], Jinhua Zhang[1], Wei Zhen[1], Yao Wang[1], Deborah J. Clegg [3], Bin Xu[2], Zhiyong Cheng[1], Ryan P. McMillan[1], Matthew W. Hulver[1], Dongmin Liu[1]

## Supplementary Figure Legends

**Supplementary Figure1. Deletion of GPR30 had no effect on metabolic phenotypes in male mice fed a HFD.** WT and KO male mice (12 wks old) with identical initial body weight were fed a HFD for 22 wks. Weekly body weight (**A**), food consumption as calorie intake (**B**), fat (**C**) and lean (**D**) mass, and non-fasting blood glucose (NFBG) levels (**E**) are shown. Data are mean ±SEM (n=8 mice/group).

**Supplementary Figure 2. There was no difference in GPR30 gene expression in white adipose tissue between male and female mice.** WT female mice and their male littermates fed a STD were euthanized at 18 wks old, and the gonadal fat was then collected for measuring mRNA levels. Data are mean ±SEM (n=4 mice/group).

**Supplementary Figure 3. GPR30 deletion had no effects on fat absorption and metabolism. A**. Fecal lipid contents from mice fed a HFD for 10 and 19 wks. **B**. Mice were fasted overnight and a bolus of olive oil (10 µl/g BW via oral gavage) was administrated, followed by measuring plasma triglyceride levels at various times. Liver triglyceride content (**C**), fasting plasma cholesterol (**D**), non-esterified fatty acids (NEFA) (E), and triglycerides (**F**) were determined. A: n=6 mice/group; B-F: n=8 mice/group. Data are mean ±SEM. *, $p<0.05$.

**Supplementary Figure 4. GPR30 KO had no effects on plasma 17β-estradiol (E2) levels and gene expression of ERα/β and adipogenic transcriptional factors in white adipose tissue.** Mice (12 wks old) were fed a HFD for 23 wks (GPR30) followed by measuring circulating E2 levels (**A**) and analyzing ER (**B**), Pparg, Cebpa, Cebpb, and Bmp (**C**) mRNA levels mRNA levels in gonadal fat. Data are mean ±SEM (n=8 mice/group).

**Supplementary Figure 5. Full-length gel image corresponding to Figure 6C-D.** 1: GPR30 in differentiated fat cells; 2: GPR30 in stromal vascular fraction (SVF) cells; 3: 18S rRNA in differentiated fat cells; 4: 18S rNNA in SVF cells.

**Supplementary Figure 1**

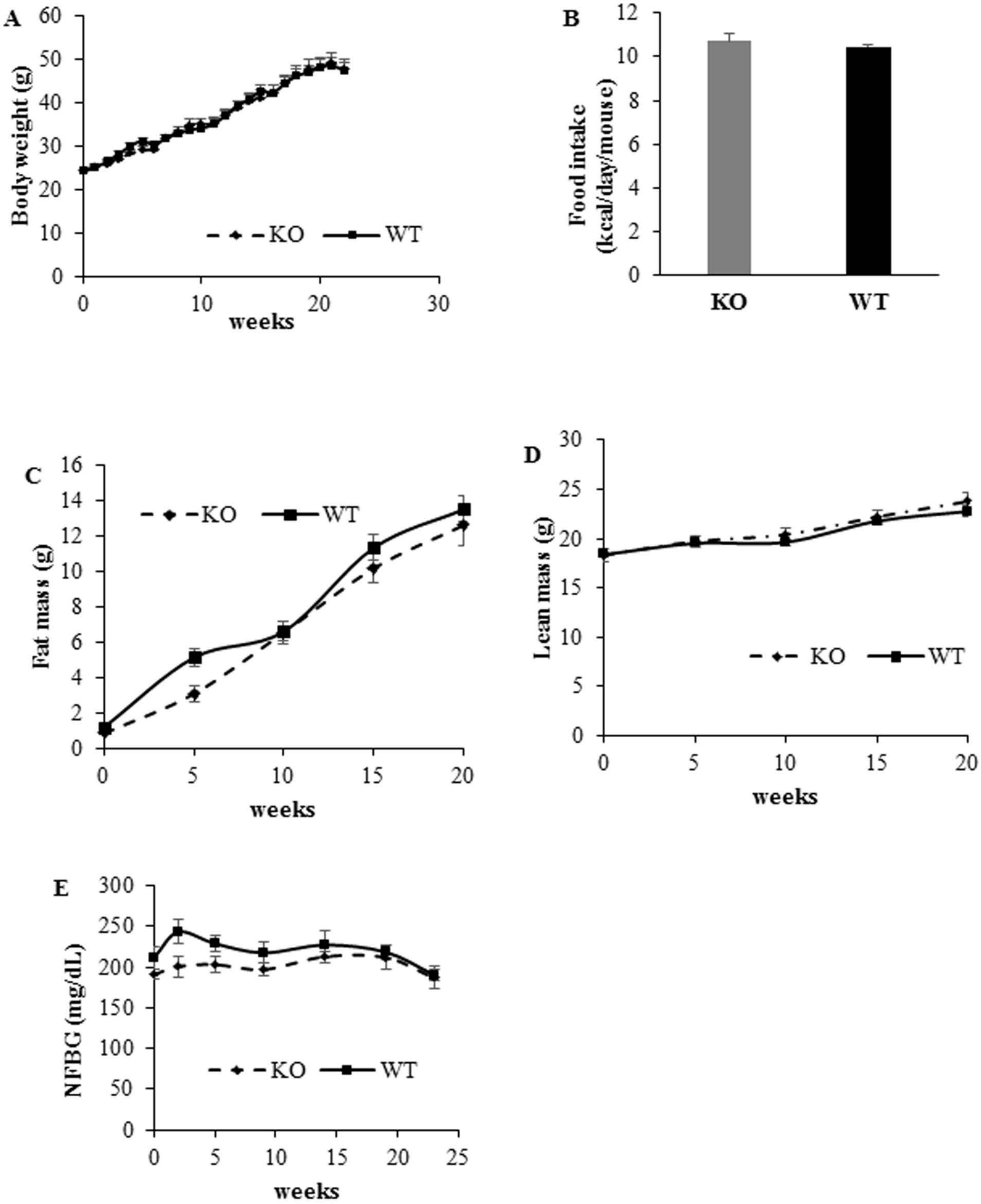

**Supplementary Figure 2**

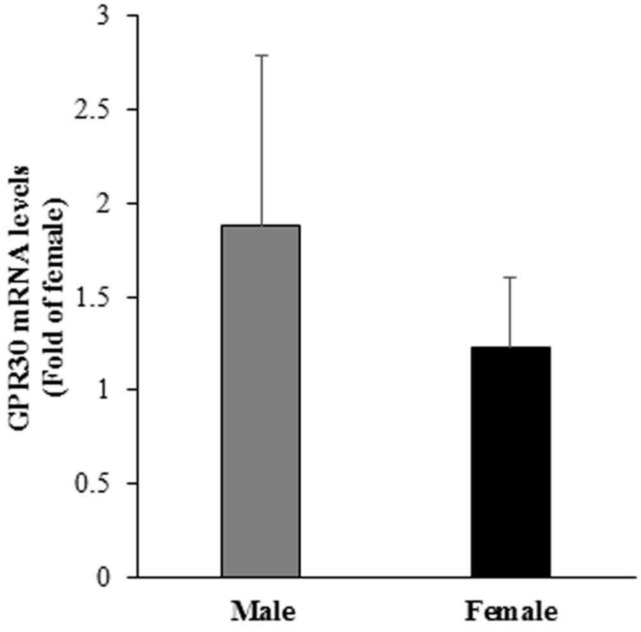

# Supplementary Figure 3

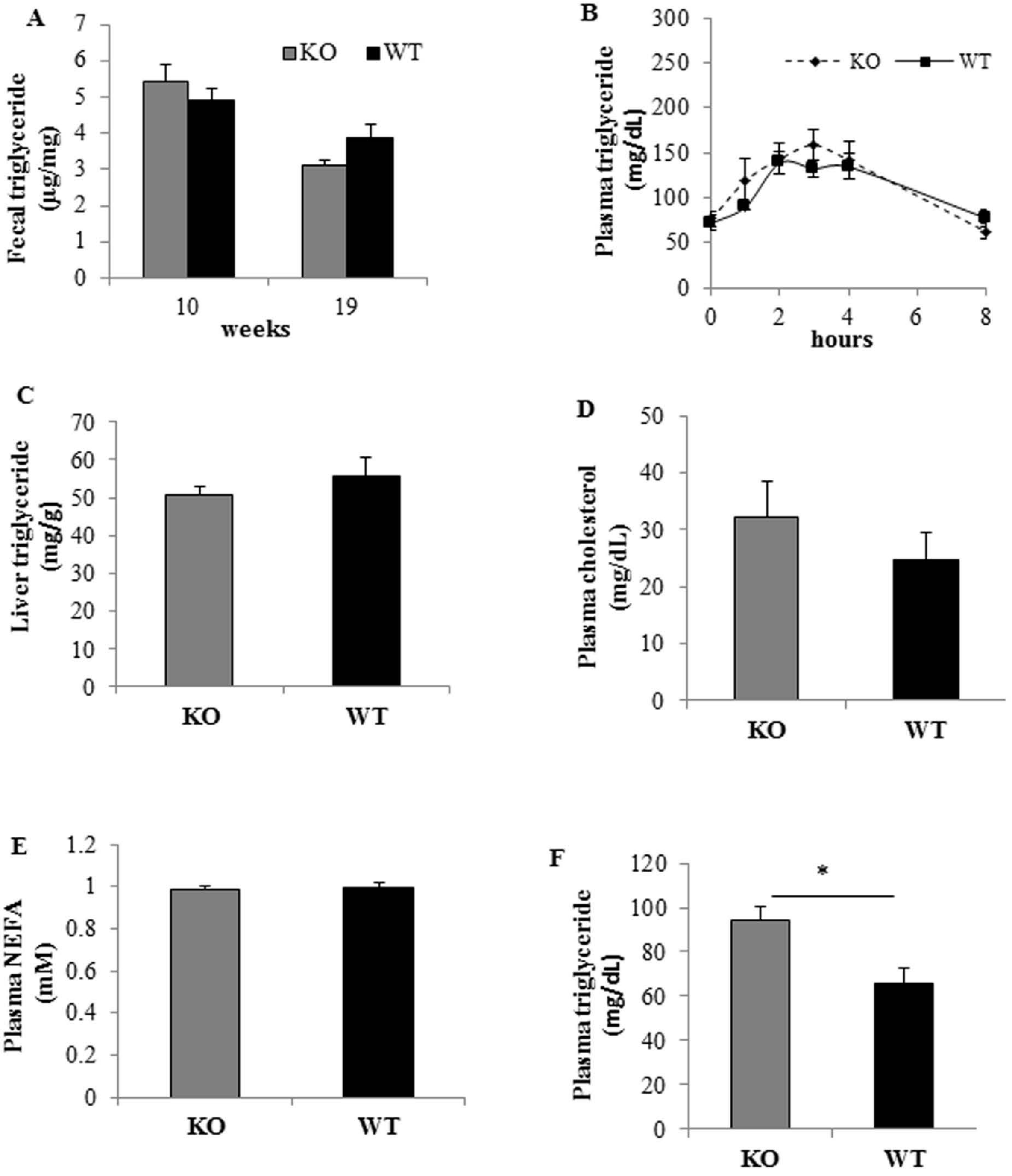

## Supplementary Figure 4

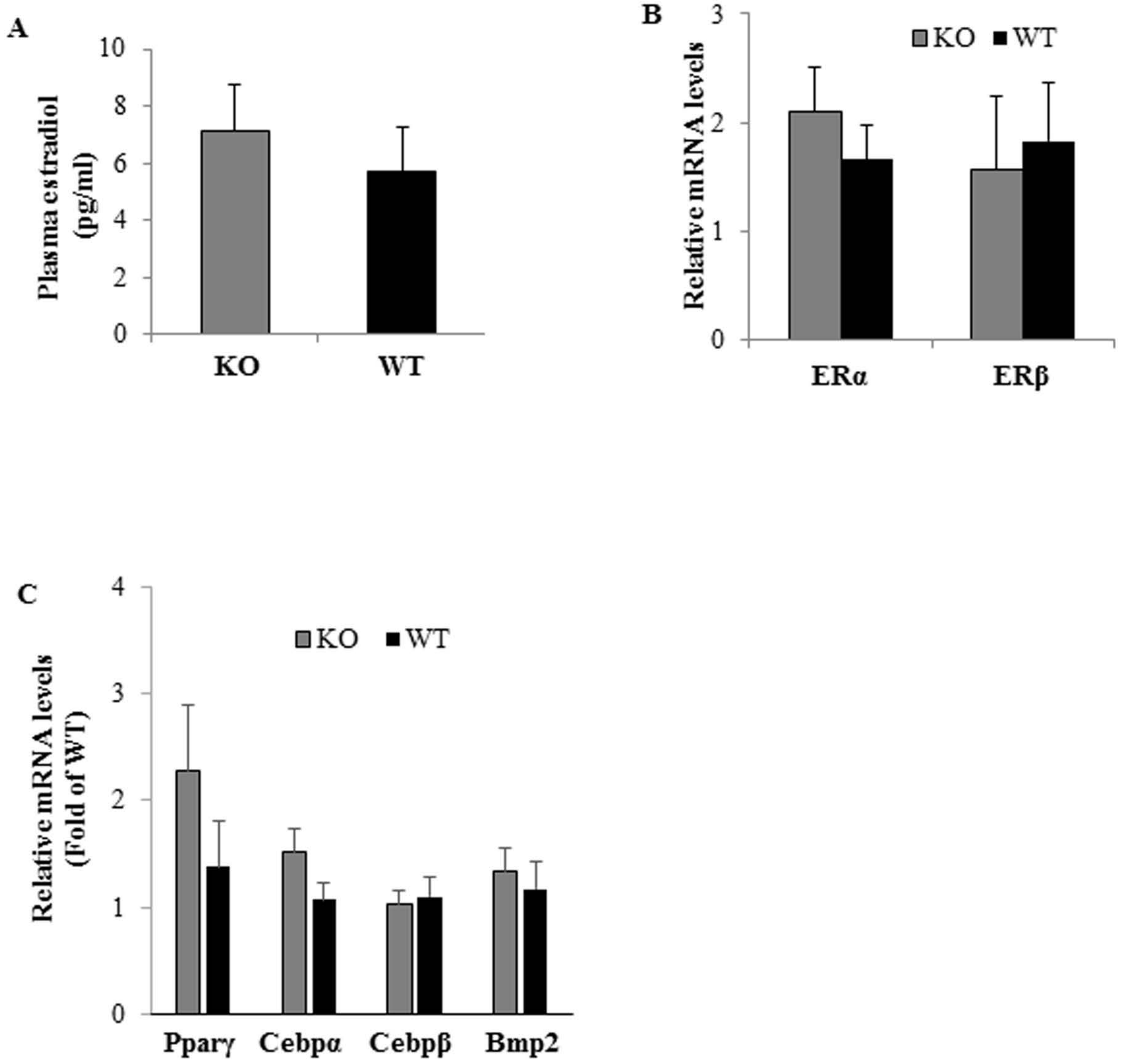

**Supplementary Figure 5**

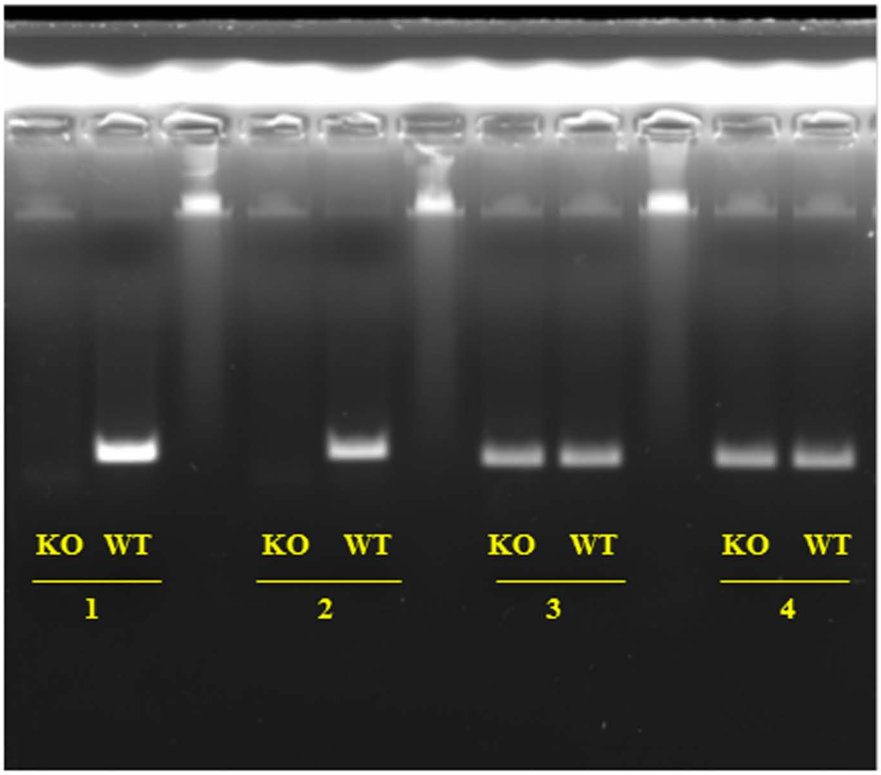

Supplement: Supplementary Information [file srep34302-s1.pdf]
